# Usnic Acid-Mediated Exchange of Protons for Divalent Metal Cations across Lipid Membranes: Relevance to Mitochondrial Uncoupling

**DOI:** 10.3390/ijms232416203

**Published:** 2022-12-19

**Authors:** Tatyana I. Rokitskaya, Alexander M. Arutyunyan, Ljudmila S. Khailova, Alisa D. Kataeva, Alexander M. Firsov, Elena A. Kotova, Yuri N. Antonenko

**Affiliations:** 1 Belozersky Institute of Physico-Chemical Biology, Lomonosov Moscow State University, 119991 Moscow, Russia; 2 Faculty of Chemistry, Lomonosov Moscow State University, 119991 Moscow, Russia

**Keywords:** usnic acid, bilayer lipid membrane, mitochondrial uncoupler, protonophore, divalent metal cation/proton exchange, circular dichroism

## Abstract

Usnic acid (UA), a unique lichen metabolite, is a protonophoric uncoupler of oxidative phosphorylation, widely known as a weight-loss dietary supplement. In contrast to conventional proton-shuttling mitochondrial uncouplers, UA was found to carry protons across lipid membranes via the induction of an electrogenic proton exchange for calcium or magnesium cations. Here, we evaluated the ability of various divalent metal cations to stimulate a proton transport through both planar and vesicular bilayer lipid membranes by measuring the transmembrane electrical current and fluorescence-detected pH gradient dissipation in pyranine-loaded liposomes, respectively. Thus, we obtained the following selectivity series of calcium, magnesium, zinc, manganese and copper cations: Zn^2+^ > Mn^2+^ > Mg^2+^ > Ca^2+^ >> Cu^2+^. Remarkably, Cu^2+^ appeared to suppress the UA-mediated proton transport in both lipid membrane systems. The data on the divalent metal cation/proton exchange were supported by circular dichroism spectroscopy of UA in the presence of the corresponding cations.

## 1. Introduction

Usnic acid (UA) has long been a popular fat-burning pill because of its ability to cause the dissipation of the proton motive force in mitochondria [1,2,3,4,5,6], similar to conventional mitochondrial uncouplers [7,8,9]. In contrast to the most common—and rather compromised, due to its side effects—chemical uncoupler 2,4-dinitrophenol (DNP), UA is a natural compound, a secondary lichen metabolite known for its numerous therapeutically beneficial modalities [4,10,11,12,13,14,15]. Although UA has been shown to induce a proton-selective current across a planar bilayer lipid membrane (BLM) [16] typical of protonophoric mitochondrial uncouplers [17], our previous studies revealed its unique properties; in particular, its ability to mediate an electrogenic proton/divalent cation exchange across a BLM [18], e.g., three protons per one magnesium cation [19]. According to the literature [20], UA is involved in heavy metal accumulation detoxification by lichens. However, the UA-mediated transport of divalent metal cations across natural and artificial membranes has not been explored in detail. In this work, we compared the potency of calcium, magnesium, zinc, manganese and copper cations in promoting a UA-mediated proton flow across both a planar and a vesicular BLM, thereby examining the selectivity of the calcium, magnesium, zinc, manganese and copper cations/proton exchange elicited by UA. The following order of UA transport activity was obtained: Zn^2+^ > Mn^2+^ > Mg^2+^ > Ca^2+^ >> Cu^2+^. Interestingly, Cu^2+^ was found to inhibit the UA-mediated proton transport. The results on the transport through the membranes were supplemented by circular dichroism (CD) spectroscopy data, which showed the binding of the metal cations to UA in methanol.

## 2. Results and Discussion

### 2.1. Impact of Divalent Metal Cations on a UA-Induced Electrical Current across a Planar BLM

**Figure 1:**
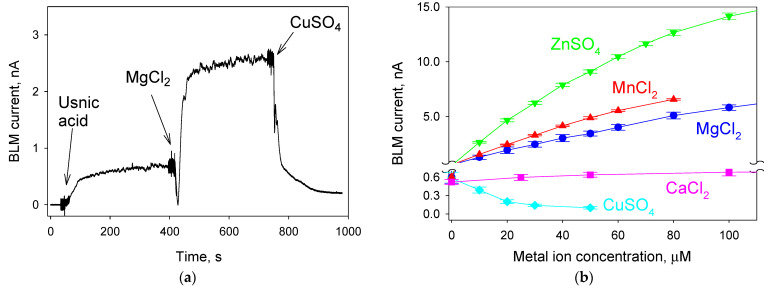
(**a**) Effect of magnesium ions (20 µM) on the induction of electrical current through BLM by usnic acid (UA, 20 µM) and the inhibitory effect of copper ions (20 µM); (**b**) dependence of the UA-mediated electrical current through BLM on the concentration of calcium, magnesium, manganese, copper and zinc ions. The solution was 10 mM Tris, 10 mM MES, 10 mM β-alanine and 10 mM KCl with a pH of 6.0. BLM voltage was 30 mV. BLM was created from DPhPC. The data in panel (**b**) are represented as the mean ± SE, with *n* = 3.

Figure 1a shows a track of an electrical current across a BLM induced by the addition of 20 μM UA. Without the added divalent cations, the non-zero magnitude of the current was obviously due to the presence of traces of calcium ions in the buffer solution. The addition of EDTA suppressed the current to a zero level (Chart S1 in the Supplementary Materials), in agreement with our previous data [6]. The addition of 20 µM MgCl_2_ led to a substantial increase in the current, reaching 2.5 nA within 2 min of the stirring of the membrane-bathing solution. Under these conditions, a UA-mediated current, depending on both the pH and Mg^2+^ concentration, resulted from the electrogenic Mg^2+^/3H^+^ exchange [19]. The subsequent addition of 20 µM CuSO_4_ caused a substantial decrease in the BLM current, probably due to the strong binding of Cu^2+^ to UA, interfering with its ability to transport the cations. In the control experiments, the addition of 20 µM (or more) K_2_SO_4_ did not affect the UA-mediated BLM current, thereby proving that it was Cu^2+^ that inhibited the transport (Chart S2 in the Supplementary Materials). Figure 1b displays the dependence of the UA-mediated BLM current on the concentration of divalent metal cations in the solution. It is seen that the cations stimulated the current in the following series: Zn^2+^ > Mn^2+^ > Mg^2+^ > Ca^2+^. The addition of Cu^2+^ actually led to its suppression (Figure 1b, blue points).

### 2.2. UA-Mediated Alkalization inside Liposomes in the Presence of Divalent Metal Cations

**Figure 2:**
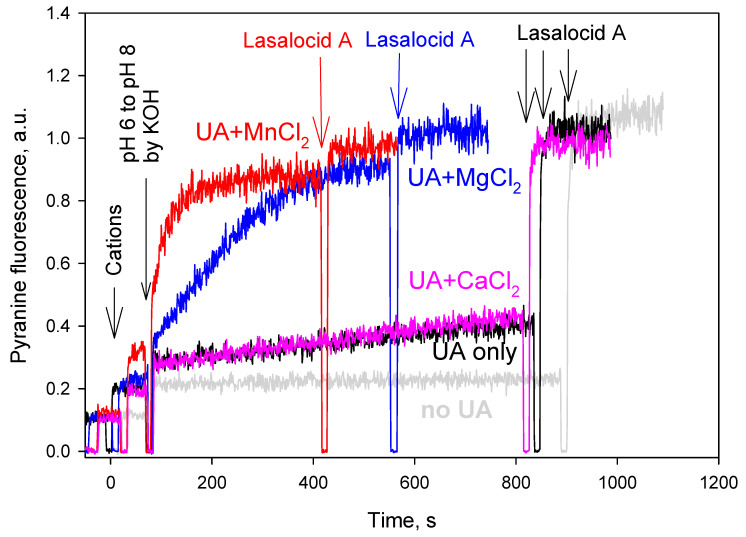
Effect of divalent metal cations (1 mM each) on usnic acid-mediated proton fluxes through liposomes loaded with the pH probe pyranine. The concentration of usnic acid was 30 µM. The inner liposomal pH was estimated from the pyranine fluorescence intensities measured at 505 nm upon excitation at 455 nm. Lasalocid A (1 µM) was added at the end of each track to equilibrate the pH. Lipid concentration was 20 µg/mL. Other conditions: see Materials and Methods section. At the zero-time moment, divalent metal cations were added in the form of chlorides at a concentration of 1 mM. The proton flux was stimulated by an alkaline pH shift from pH 6 to pH 8 at t = 75 s by the addition of the previously determined aliquot of KOH. The measurements were made at 15 °C in order to decrease the unspecific proton leak in liposomes.

To confirm the divalent cation preference of UA by an alternative experimental technique, we monitored the pH gradient resulting from the cation–proton exchange activity of UA in the pyranine-loaded liposomes. The addition of 1 mM MnCl_2_ (red curve in Figure 2), 1 mM MgCl_2_ (blue curve) or 1 mM CaCl_2_ (pink curve) to the buffer solution (pH 6.0) outside the liposomes containing 30 µM UA caused alkalization inside the liposomes, manifesting in an increase in the pyranine fluorescence intensity as a result of the influx of the divalent cations and the efflux of the protons from the liposomes (Figure 2). The subsequent addition of KOH at 75 s, shifting the external pH from 6 to 8, led to a substantial stimulation of the alkalization inside the liposomes. The alkalization was the most pronounced in the case of MnCl_2_ (red track), intermediate in the case of MgCl_2_ (blue track) and small in the case of CaCl_2_ (pink track). The black track displayed a weak alkalization of the liposomal lumen induced by UA without the addition of divalent cations. No alkalization took place inside the liposomes in the absence of UA, thereby showing that there was no proton leakage from the liposomes without UA (grey track). In the control experiments without UA, even 1 mM Mn^2+^ did not elicit a proton leakage (Chart S3 in the Supplementary Materials). We have not presented the data of similar experiments with ZnCl_2_ and CuCl_2_ because these cations caused a strong quenching of the pyranine fluorescence (Chart S4 in the Supplementary Materials). Therefore, the liposome system showed the following series of UA cation selectivity: Mn^2+^ > Mg^2+^ > Ca^2+^.

### 2.3. UA-Mediated Efflux of Divalent Cations from Liposomes

**Figure 3:**
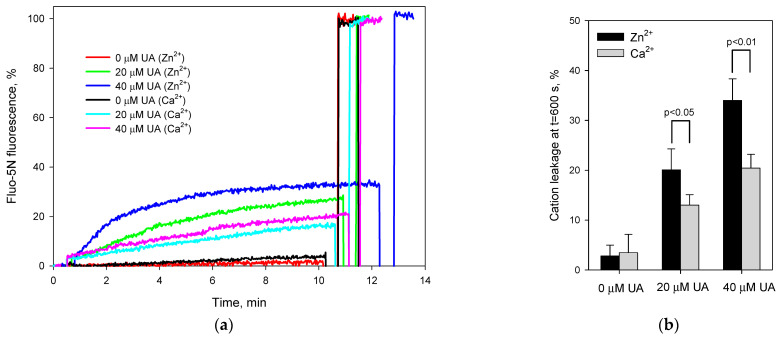
Efflux of Ca^2+^ or Zn^2+^ from Ca^2+^- or Zn^2+^-loaded liposomes induced by usnic acid. Panel (**a**) displays time courses of the cation efflux from the liposomes, panel (**b**) shows a histogram of the cation efflux after 10 minutes of the efflux. The data were normalized to the maximal fluorescence level achieved after the addition of 100 nM ionomycin. The concentrations of Ca^2+^ or Zn^2+^ ions were estimated from the Fluo-5N fluorescence intensities measured at 520 nm upon excitation at 495 nm. Lipid concentration was 20 µg/mL; T = 25 °C. Other conditions: see Materials and Methods section.

To confirm the divalent cations/proton exchange mediated by UA on the liposomes, we measured the Zn^2+^ and Ca^2+^ efflux from the liposomes loaded with these cations by using Fluo-5N as a fluorescent probe. Figure 3a shows the kinetics of the Fluo-5N fluorescence intensity after the addition of UA at a concentration of 20 μM (green and cyan curves) or 40 μM (blue and pink curves). The data were normalized to the level of the complete efflux of the cations elicited by the addition of the conventional calcium ionophore ionomycin at the end of each track. The efflux of zinc ions was faster than that of the calcium ions at all concentrations of UA tested (Figure 3b). The preference of Zn^2+^ over Ca^2+^ was in agreement with the data from both the planar BLM (Figure 1) and the pyranine-loaded liposomes (Figure 2).

### 2.4. Effect of Divalent Metal Cations on the CD and Absorbance Spectra of UA

**Figure 4:**
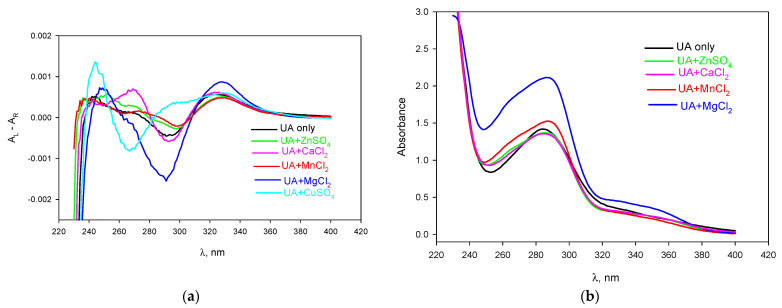
CD (**a**) and absorbance (**b**) spectra of UA (50 µM) in the absence (black curve) and in the presence of 1 mM calcium (pink), magnesium (blue), zinc (green), manganese (red) and copper (cyan) ions in methanol. The concentration of CuSO_4_ was 0.1 mM.

To examine the formation of metal–UA complexes, we measured the CD and UV absorbance spectra of UA in the presence of divalent metal cations in methanol, enabling both lipophilic UA and hydrophilic inorganic salts to be dissolved. The CD spectrum of UA in methanol exhibited two positive bands peaking at about 250 nm and 330 nm and a negative band peaking at about 290 nm (black curve in Figure 4a) in agreement with the literature data [21]. Interestingly, the CD spectrum of UA in chloroform is known to be substantially different: there is a positive broad band around 260 nm and two weak negative bands at both sides [22]. In line with [21], we found that Cu^2+^ severely altered the CD spectra of UA, leading to the appearance of a negative 270 nm band at low Cu^2+^ concentrations, which indicated the formation of a tight UA–Cu^2+^ complex (cyan curve in Figure 4a), as suggested by the effect of Cu^2+^ on the BLM current (Figure 1). Of note, the strong binding of Cu^2+^ to UA might be involved in a high copper toxicity to lichens [23]. Other cations did not distort the CD spectrum of UA; an increase in both the positive 330 nm band and the negative 290 nm band was observed in the CD spectrum of UA upon the addition of the cations (Figure 4a). Figure 4b shows the UV spectra of UA in the presence of a 1 mM concentration of Zn^2+^, Mn^2+^, Mg^2+^ and Ca^2+^. The addition of the cations led to an increase in the absorbance at 290 nm; the effect of Mg^2+^ was the most pronounced (blue curve). Overall, the spectral data pointed to the chelation of the divalent metal cations by usnic acid.

Of note, we previously failed to detect a UA-mediated efflux of Zn^2+^ from liposomes loaded with this cation [18], which was probably due to the low Zn^2+^ sensitivity of the fluorescence technique used in the experiments. Here, we found the zinc cations to be more effective in promoting he UA-mediated proton transport than the magnesium and calcium cations, thus indicating the higher selectivity of the UA-induced divalent cation/proton exchange for Zn^2+^ than for Mg^2+^ and Ca^2+^.

As aforementioned, UA is used as a mitochondrial uncoupler to stimulate metabolism. This study, together with our previous results [6,18,19], indicated that UA induced a proton transfer into the mitochondria in exchange for the efflux of the divalent cations from the mitochondria, thereby suggesting that UA-mediated uncoupling should depend on the concentration of calcium and magnesium cations in the mitochondrial matrix. This should be kept in mind when studying the action of UA in physiological experiments as both the magnesium content and, to an even greater extent, the calcium content inside the mitochondria depend on the physiological conditions of cells [24,25,26].

## 3. Materials and Methods

### 3.1. Materials

Most chemicals, including (+)-usnic acid (UA) and diphytanoylphosphatidylcholine (DPhPC), were from Sigma-Aldrich, St. Louis, MO, USA.

### 3.2. Planar Lipid Bilayers

A bilayer lipid membrane (BLM) was formed using the brush technique [27] from a 2% decane solution of DPhPC on a 0.8 mm hole in a Teflon partition that separated the experimental cell into two 3 mL compartments. The electrical current through the BLM was measured with two Ag/AgCl electrodes placed into the solutions on the opposite sides of the BLM via agar bridges by using a Keithley 428 amplifier (Cleveland, OH, USA).

### 3.3. Pyranine-Loaded Liposomes

To measure the proton efflux from the liposomes, we prepared large unilamellar vesicles loaded with the pH-sensitive fluorescent dye pyranine and used the method described in [28] with slight modifications. The liposomes were created from a mixture of 2.5 mg POPC, 1 mg POPG and 1.5 mg cholesterol dissolved in chloroform. The pyranine fluorescence intensity was measured at 505 nm upon excitation at 455 nm with a Panorama Fluorat 02 spectrofluorometer (Lumex, Saint-Petersburg, Russia). At the end of each recording, 1 µM lasalocid A was added to dissipate the remaining pH gradient. To prevent the formation of a proton diffusion potential, all the measurements were performed in the presence of 10 nM valinomycin.

### 3.4. Ca^2+^- and Zn^2+^-Loaded Liposomes

The preparation of liposomes loaded with 100 mM CaCl_2_ or ZnSO_4_ and the measurements of the Ca^2+^ (Zn^2+^) efflux from the liposomes were performed as described in [18]. The outside Ca^2+^ and Zn^2+^ were assayed by measuring the fluorescence intensity of the Ca^2+^ (Zn^2+^)-sensitive fluorophore Fluo-5N at 520 nm upon excitation at 495 nm with a Solar CM2203 spectrofluorometer (Minsk, Belarus). To remove the contribution of 3–5 µM Ca^2+^, always present in the medium outside the liposomes, to the Fluo-5N fluorescence, we added an aliquot of 1 mM EDTA before the addition of UA.

### 3.5. Circular Dichroism Spectra of Metal–Usnic Acid Complexes in Methanol

The electronic circular dichroism (CD) spectra were recorded on a Chirascan CD spectrometer (Applied Photophysics, Leatherhead, UK) using a 1 cm quartz cuvette.

## Supplementary Materials

The following supporting information can be downloaded at: https://www.mdpi.com/article/10.3390/ijms232416203/s1.
