# Supplementary material for: Usnic Acid-Mediated Exchange of Protons for Divalent Metal Cations across Lipid Membranes: Relevance to Mitochondrial Uncoupling
Source: Int J Mol Sci. 2022 Dec 19;23(24):16203. doi: 10.3390/ijms232416203 (PMC9783568; doi:10.3390/ijms232416203)
Supplement: Supplementary file 1 [file ijms-23-16203-s001.zip › ijms-2040506-supplementary.pdf]

# Usnic acid-mediated exchange of protons for divalent metal cations across lipid membranes: relevance to mitochondrial uncoupling

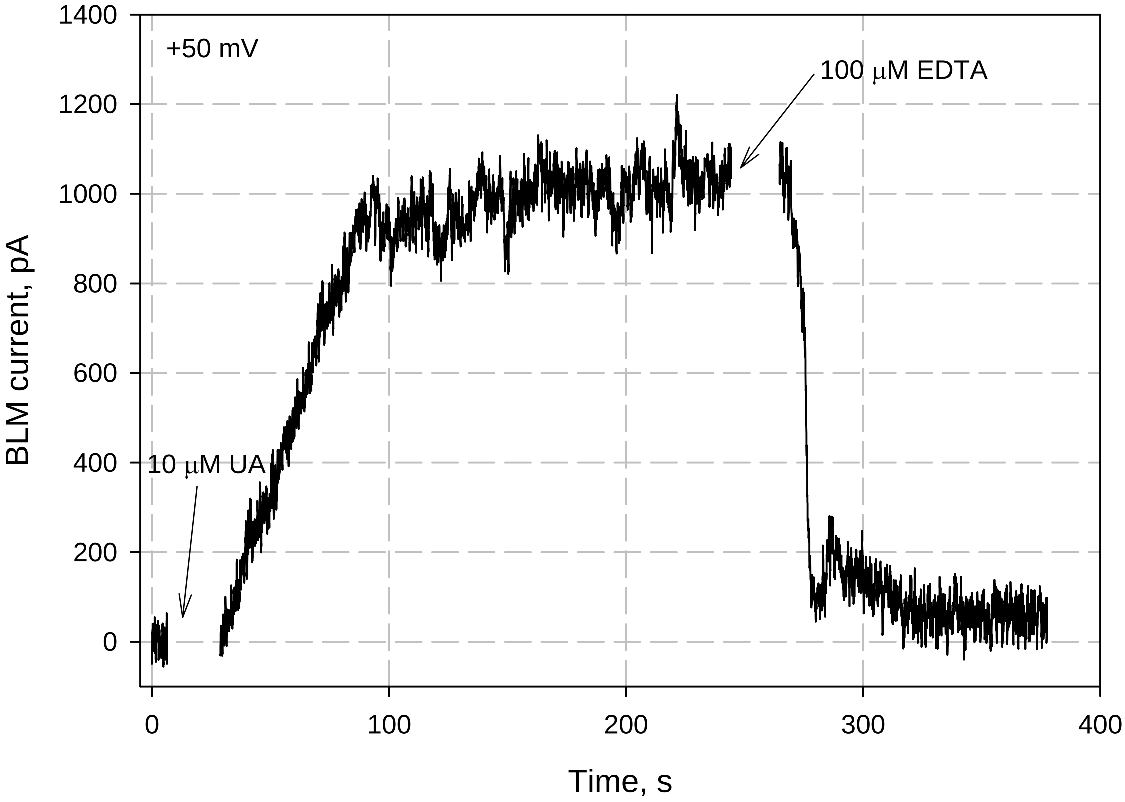


**Chart S1.** Effect of calcium chalator EDTA (100 μM) on the induction of electrical current through BLM by usnic acid (UA, 10 μM). The solution was 10 mM Tris, 10 mM MES, 10 mM β-alanine, 10 mM KCl, pH 7.0. BLM voltage was 50 mV. BLM was made from DPhPC.

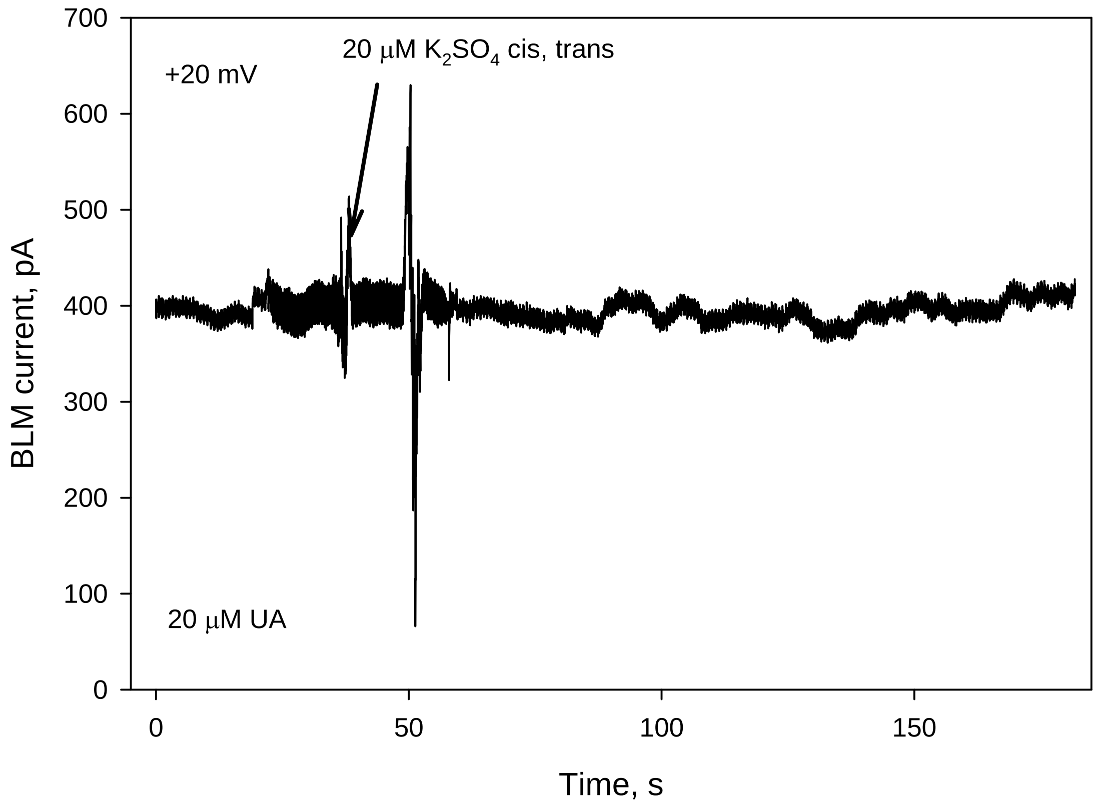


**Chart S2.** Effect of $K_2SO_4$ (20 µM) on the usnic acid-mediated electrical current through BLM (UA, 20 µM). The solution was 10 mM Tris, 10 mM MES, 10 mM β-alanine, 10 mM KCl, pH 6.0. BLM voltage was 20 mV. BLM was made from DPhPC.

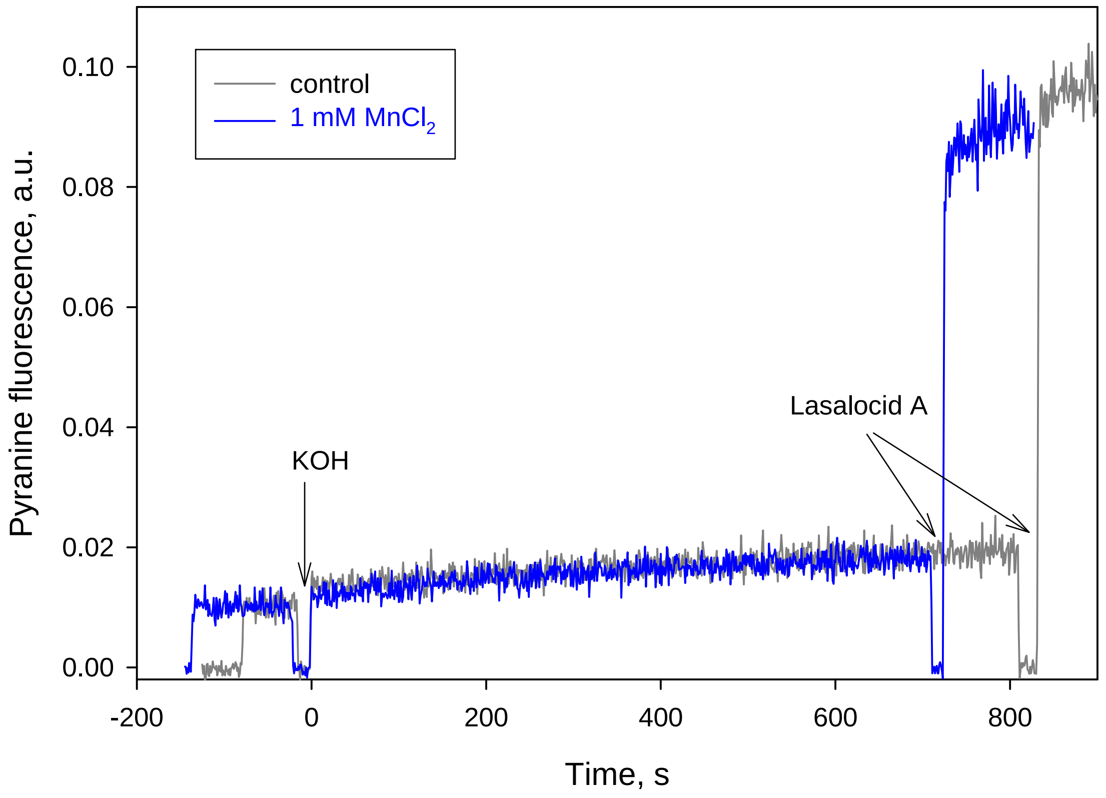


**Chart S3.** Effect of $MnCl_2$ (1 mM) on the control proton fluxes (in the absence of usnic acid) through liposomes loaded with the pH probe pyranine. The inner liposomal pH was estimated from the pyranine fluorescence intensities measured at 505 nm upon excitation at 455 nm. 1 µM lasalocid A was added at the end of each track to equilibrate the pH. Lipid concentration was 20 µg/ml. Other conditions: see Materials and Methods.

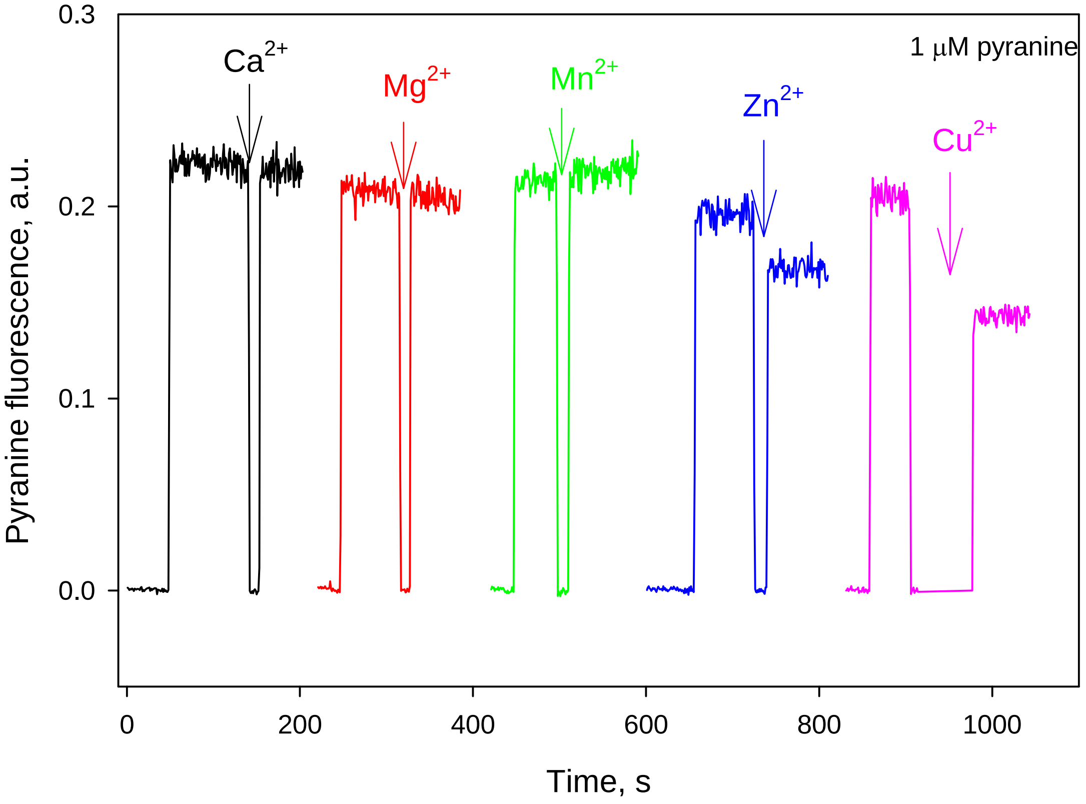


**Chart S4.** Quenching of Pyranine fluorescence (1 µM) in aqueous solution by 1 mM of different divalent cations without liposomes. The fluorescence was measured at 505 nm upon excitation at 455 nm. The solution was 100 mM KCl, 20 mM MES, 20 mM MOPS, 20 mM Tricine titrated with KOH to pH 6.0.
